# Successful closure of chronic recurrent Enterocutaneous fistula with a concurrent over‐the‐scope closure and a stent placement

**DOI:** 10.1002/ccr3.2652

**Published:** 2020-01-20

**Authors:** Abdulfatah Issak, Mustafa Musleh

**Affiliations:** ^1^ Department of Gastroenterology Wright State University Boonshoft School of Medicine Dayton OH USA

**Keywords:** Enterocutaneous fistula, gastrointestinal perforations, nonsteroidal anti‐inflammatory drugs, perforated peptic ulcer, perforations

## Abstract

Gastrointestinal ulcers and perforations can occur as a complication of nonsteroidal anti‐inflammatory drugs (NSAID). Emerging endoscopic technologies can be utilized to successfully intervene perforations that fail surgical intervention. We report a case of perforated duodenal ulcer that failed surgical intervention and, however, was successfully closed with over‐the‐scope clip (OTSC) closure with concomitant placement of fully covered stent.

## Graphical abstract

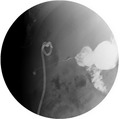

## 1 INTRODUCTION

Gastrointestinal viscus perforation is associated with high morbidity and mortality. Traditionally, surgery has been the primary intervention; however, recent literature indicates increasing role for new emerging endoscopic technologies leading to minimally invasive intervention of perforated peptic ulcers and fistulae.

## 2 CASE REPORT

**Figure 1:**
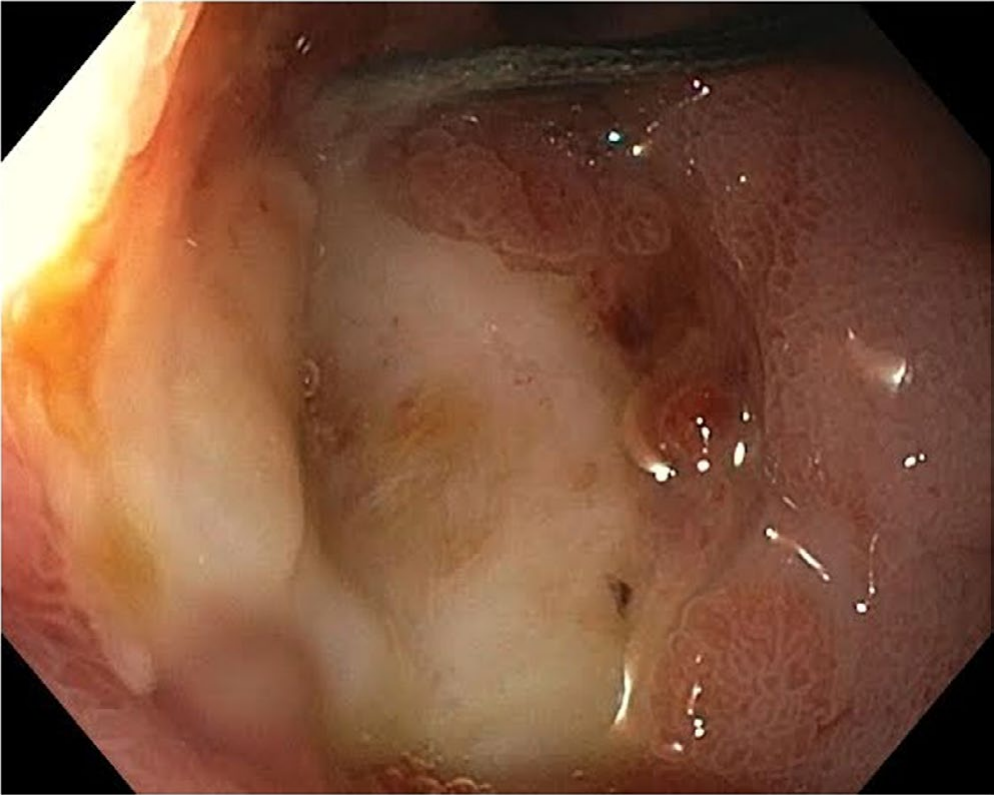
Duodenal ulcer

**Figure 2:**
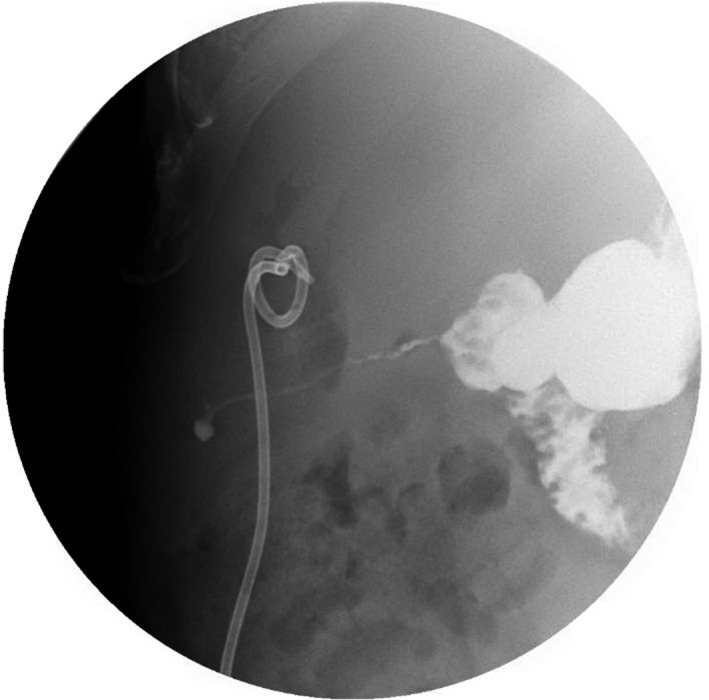
Enterocutaneous fistula from the duodenal bulb to the abdominal wall

**Figure 3:**
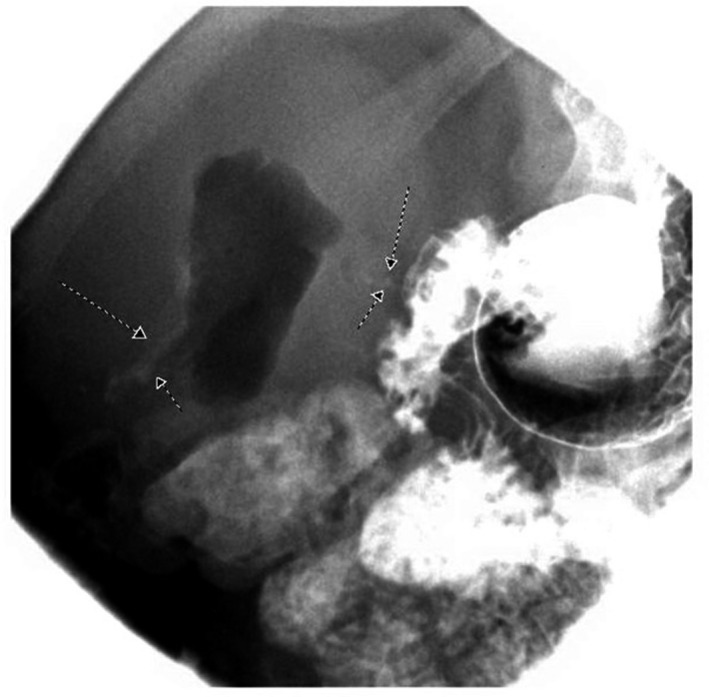
Postsurgical fistulectomy with recurrent fistula

**Figure 4:**
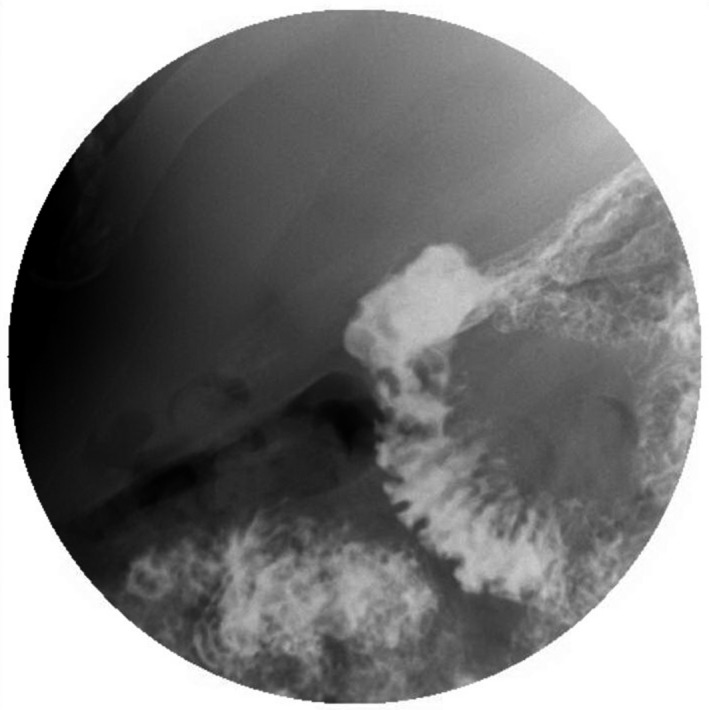
Small bowel flow through after over‐the‐scope clip and stent closure with resolution of the fistula

A 58‐year‐old Caucasian male patient on a long‐term nonsteroidal anti‐inflammatory drug (NSAID) for chronic arthritis presented with several hours of severe abdominal pain, nausea, and vomiting. On physical examination, he was found to have distended abdomen that is severely painful to deep palpation. Rebound tenderness and involuntary guarding were noted. Pain was most pronounced in bilateral upper quadrants and epigastric area. He had signs of sepsis but hemodynamically stable (temperature of 101.3 Fahrenheit, blood pressure of 112/70 mm mercury, heart rate of 102 beats/min, respiratory rate of 23 breaths/min, oxygen saturation of 96% in room air, white blood cell counts of 19 × 10^9^ cells per litter, lactate of 2.2 millimole/L). Computed tomography (CT) scan demonstrated free air and fluid in the abdomen. He had emergent exploratory laparoscopy, which showed a perforated duodenal ulcer (Figure 1) and underwent Graham patch closure. Patient was discharged home on hospital day 9 tolerating regular diet. Three weeks later, he presented with nausea, vomiting, and poor oral intake. On examination, he was noted to have cutaneous fluctuance in the right abdomen. CT scan of abdomen demonstrated abscess in the right upper abdominal quadrant. He was then taken to the operating room for abdominal wound exploration and was found to have intra‐abdominal abscess with small fistulous tract to the right abdominal wall (Figure 2). The entire fistulous tract was resected. He was discharged home in two weeks. A month later, patient was readmitted with recurrent abdominal abscess and recurrent discharge from his cutaneous fistula (Figure 3). Imaging showed recurrent intra‐abdominal abscess, which was treated with antibiotics and percutaneous Jackson‐Pratt (JP) drain placement. The patient declined a repeat surgical intervention and opted for an endoscopic approach. An esophagogastroduodenoscopy (EGD) was done, which showed a 2‐ to 3‐millimeter persistent fistulous opening in the inferior wall of the duodenal bulb. Significant duodenal bulb edema was present, but no fibrosis was noted. Fistula opening was then closed using over‐the‐scope clip (OTSC; Ovesco, type T, size 11 with 3 mm cap depth). However, one day later a CT scan of abdomen showed the clip had fallen and was present in the splenic flexure. EGD was repeated, and endoscopic closure was reattempted using an over‐the‐scope clip OTSC (Ovesco, type T, size 11, with 6 mm cap depth) which was applied to fistula opening successfully. At the same time, a fully covered metal stent was deployed through the scope and under fluoroscopic guidance into the duodenum bridging the leak area (Niti‐S 20 mm diameter and 60 mm long, product of TaeWoong Medical). Distal end of stent was placed proximal to the papilla. The stent was anchored in place with two end clips to the gastric wall in an attempt to prevent stent migration. A pureed diet was started in 5 days. A repeat upper gastrointestinal (GI) series prior to discharge showed stent and clip in good position with no evidence of leak, and the patient was discharged home on a pureed diet for an additional one week. The patient had an uneventful course and had a repeat EGD 6 weeks postprocedure for stent removal, which showed the stent had migrated into the stomach, which was removed. A clean‐base duodenal ulcer was noted at the duodenal bulb but without any visible openings. A small bowel follow‐through few days later showed no evidence of fistula or leak (Figure 4). Patient remains asymptomatic without recurrence followed up to 2 years postprocedure.

## 3 DISCUSSION

Four million people suffer from peptic ulcer disease (PUD) annually around the world.^1^ Of these, perforation is reported to occur in 2%‐15% with an associated mortality in the range of 10%‐30%.^1^, ^2^, ^3^ Risk factors for PUD include Helicobacter pylori infection, nonsteroidal anti‐inflammatory drug (NSAID) use, Zollinger‐Ellison syndrome, steroid use, and concurrent anticoagulant.^4^, ^5^ NSAIDs inhibit the production of mucosal prostaglandins, which serve as a protective mechanism against gastroduodenal erosions and ulcerations.^6^ The risk of PUD and its complications including bleeding, perforation, and death are increased approximately fourfold in NSAID users.^6^, ^7^

The management of perforated PUD presents a challenge for clinicians. Traditionally, surgical approach ranging from simple closure of the perforation to definitive ulcer‐curative procedure, usually vagotomy with antrectomy, was the primary intervention.^8^ This, however, entails an invasive surgical procedure, which carries increased morbidity and mortality compared with nonoperative approach.^8^, ^9^ Those undergoing an ulcer‐curative procedure are associated with increased operative mortality of 1%‐2% and postoperative sequelae such as anastomotic leak, gastric stasis, dumping syndrome, diarrhea, and bilious vomiting.^8^, ^10^, ^11^ The nonoperative approach with intravenous proton‐pump (PPI) inhibitor or intravenous H2 antagonist is considered in selective patients without pneumoperitoneum on initial presentation.^8^ This nonoperative approach is limited by 35% longer hospital stay and poor outcome in patients over 70 years of age.^11^

The emerging endoscopic technologies are allowing gastroenterologists to utilize more conservative endoscopic approaches. An endoscopic approach has the advantages of low morbidity and mortality, shorter hospital stays.^12^ The OTSC (Ovesco, Tübingen, Germany) is an innovative device developed for the closure of small mural defects, perforations, anastomotic leaks, bleeding ulcers, and resection of small tumors. Several case reports and small case series in the literature indicate a successful closure of perforated/bleeding gastrointestinal viscus with OTSC system.^13^, ^14^, ^15^ The overall success rate of the OTSC system depends on the expertise, size, and location of gastroesophageal lesions and ranges from 66% to 75% in one small case series.^16^ The factors intrinsic to fistulas such as fibrosis can additionally impede the success of the OTSC system. It is considered to be ideal for high‐risk bleeding ulcers and for closing small (<15 mm), soft, and not fibrotic fistulas.^16^

Unique to this case is the concurrent placement of the OTSC system and a stent over the fistula. The intent of the stent placement was to provide increased leverage and prevent the OTSC from migrating as happened on the initial attempt. The successful fixation of esophageal self‐expandable metal stents (SEMS) with the OTSC system to prevent migration has been reported in the literature.^17^ However, there is a paucity of literature regarding the concomitant use of the OTSC system and a stent to close gastrointestinal fistulas that fail initial surgical and endoscopic intervention. We attribute the success of the fistula closure to both the OTSC system and the stent.

## 4 CONCLUSION

Our experience shows that the concurrent placement of the OTSC system and a stent may prove useful for perforated gastrointestinal ulcers recalcitrant to simple surgical closure, prevent the OTSC migration, and preclude need for invasive surgical intervention.

## CONFLICT OF INTEREST

None declared.

## AUTHOR CONTRIBUTIONS

AI: had the initial counter with the patient, followed the patient as an outpatient, and prepared, wrote up, and reviewed the manuscript. MM: performed all endoscopic procedures and reviewed and edited the manuscript. All authors have reviewed and approved the manuscript.
